# *FTO* Gene Polymorphism Is Associated with Type 2 Diabetes through Its Effect on Increasing the Maximum BMI in Japanese Men

**DOI:** 10.1371/journal.pone.0165523

**Published:** 2016-11-07

**Authors:** Yutaka Kamura, Minoru Iwata, Shiro Maeda, Satomi Shinmura, Yukiko Koshimizu, Hisae Honoki, Kazuhito Fukuda, Manabu Ishiki, Isao Usui, Yasuo Fukushima, Atsuko Takano, Hiromi Kato, Shihou Murakami, Kiyohiro Higuchi, Chikaaki Kobashi, Kazuyuki Tobe

**Affiliations:** 1 First Department of Internal Medicine, Faculty of Medicine, University of Toyama, Toyama, Japan; 2 Health Administration Center, University of Toyama, Toyama, Japan; 3 Laboratory for Endocrinology and Metabolism, RIKEN Center for Genomic Medicine, Yokohama, Kanagawa, Japan; 4 Department of Advanced Genomic and Laboratory Medicine, Graduate School of Medicine, University of the Ryukyus, Okinawa, Japan; 5 Division of Clinical Laboratory and Blood Transfusion, University of the Ryukyus Hospital, Okinawa, Japan; 6 Department of Internal Medicine, Asahi General Hospital, Asahi-machi, Toyama, Japan; 7 Division of Endocrinology and Metabolism, Department of Internal Medicine, Saiseikai Takaoka Hospital, Takaoka, Toyama, Japan; 8 Division of Endocrinology and Metabolism, Department of Internal Medicine, Japan Community Health care Organization Takaoka Fushiki Hospital, Takaoka, Toyama, Japan; 9 Division of Endocrinology and Metabolism, Department of Internal Medicine, Toyama Rosai Hospital, Uozu, Toyama, Japan; 10 Department of Internal Medicine, Kouseiren Itoigawa General Hospital, Itoigawa, Niigata, Japan; 11 Department of Internal Medicine, Kamiichi General Hospital, Kamiichi-machi, Toyama, Japan; Universita degli Studi Magna Graecia di Catanzaro Scuola di Medicina e Chirurgia, ITALY

## Abstract

**Aim:**

Several studies have demonstrated that polymorphisms within the fat-mass and obesity-associated gene (*FTO*) are associated with type 2 diabetes (T2D). However, whether the effects of the *FTO* locus on T2D susceptibility are independent of fat-mass increases remains controversial. To investigate this issue, we examined the association of *FTO* variants with T2D and various aspects of BMI history during adult life in a Japanese population.

**Methods:**

We genotyped SNPs within *FTO* (rs1121980 and rs1558902) in 760 Japanese patients with T2D who had reached a lifetime maximum BMI (BMI_max_) before or at the time of diagnosis and 693 control individuals with information regarding their BMI_max_.

**Results:**

The BMI_max_ showed the strongest association with T2D risk among the BMIs evaluated in this study. In the sex-combined analysis, *FTO* SNPs were not associated with any of the BMI variables or with T2D, but in sex-stratified analyses, both SNPs were significantly associated with the BMI_max_ and rs1558902 was associated with T2D in men. The association of the SNPs with T2D remained significant after adjustments for the current BMI and age, whereas the T2D association of the SNP was no longer significant after adjustments for BMI_max_ and age.

**Conclusions:**

These results suggest that the effects of *FTO* polymorphisms on T2D susceptibility in Japanese men are mediated through their effect on increasing the BMI_max_ before or at the time of diagnosis.

## Introduction

Numerous studies have reported that polymorphisms within the fat-mass and obesity-associated gene (*FTO*) are strongly associated with obesity [1–7], and obesity is a major risk factor for type 2 diabetes (T2D) [8].

Regarding the *FTO* variants-T2D association, while several studies have reported that the association between the variants and risk of T2D remained significant after adjustment for BMI, a surrogate measure of obesity, others could not confirm this finding [1,9–20]. In many studies examining this issue, the BMI measured at the time of enrollment, i.e., current BMI, which was obtained long time after the diagnosis of T2D, was used for the analyses [1,9–20]. The current BMI is a useful and convenient measure for the analyses, but it is questionable that the conditioning the association between *FTO* variants and T2D on current BMI could precisely evaluate the independency of the effect of *FTO* variants on T2D risk from their effects on influencing obesity/adiposity. In general, most subjects with T2D, especially those with East Asian ethnicity, reach their lifetime maximum BMI (BMI_max_) before or at the time of the disease onset and, after the diagnosis of T2D, lifestyle interventions such as diet and exercise therapy, and/or treatments with some anti-diabetic medicines often influence their obesity related measures, such as BMI [21–23]. In many cases, the 'current BMI' was lower than BMI_max_, and as a result, the analyses using ‘current BMI’ might underestimate the effects of obesity/adiposity on conferring susceptibility to T2D, and overestimate the genetic effects of *FTO* variants on T2D risk, producing conflicting results regarding the association of *FTO* variants with T2D [24]. Therefore, BMI_max_, if available, is considered as better variable than current BMI to understand the precise mechanisms how the *FTO* variants affect the susceptibility to T2D.

In this study, we first examined the association of the various aspects of BMI history including current BMI, BMI_max_ and BMI at age 20 (BMI_20y_), with T2D risk. Then, we examined the association between *FTO* variants and T2D with or without conditioning on various aspect of BMI history including current BMI and BMI_max_.

## Materials and Methods

### Participants

We recruited 954 patients with T2D (61.0% male; age, 65.1 ± 11.4 years [mean ± SD]; and A1c, 7.6 ± 1.3%; see S1 Table) and 779 non-diabetic control individuals (46.6% male; age, 66.9 ± 10.6 years [mean ± SD]; and A1c, 5.5 ± 0.3%; see S1 Table) including individuals in a previous report (724 T2D and 763 controls, recruited between 2007 and 2009) [25] and additional 246 individuals (230 T2D and 16 controls) who regularly attended the outpatient clinics in University of Toyama Hospital (Toyama, Japan) and Itoigawa General Hospital (Itoigawa, Japan) between January 2010 and September 2014. Among these participants, we selected 760 T2D patients (80.7%) who had reached their BMI_max_ before or at the time of diagnosis and 693 control individuals with information regarding their BMI_max_ (S1 Table and Table 1). The inclusion criteria for non-diabetic controls were as follows: (1) >50 years of age, (2) HbA1c values <6.0%, (3) no family history of type 2 diabetes in first- and second-degree relatives, and (4) no past history of a diagnosis of diabetes as previously described [25]. Diabetes was diagnosed based on the 1998 American Diabetes Association Criteria [26]. The exclusion criteria for the cases with diabetes were individuals with diabetes caused by (1) liver dysfunction, (2) steroids and other drugs that might increase glucose levels, (3) malignancy, (4) monogenic disorders known to cause diabetes, and (5) individuals who tested positive for anti-GAD antibody, as previously described [25]. The clinical characteristics of the participants are shown in Table 1.

**Table 1:** Clinical characteristics of the study subjects.

	Sample size (case/control)	Type 2 diabetes	Control	*P* value
n		760	693	
Sex (M/F)		455/ 305	334/ 359	<0.0001^*^
Age at the time of examination (years)	760/693	64.6±11.5	66.5±10.8	<0.001
		(28~94)	(50~100)	
Duration of diabetes (years)	760/-	13.2±9.4	-	
Age at diagnosis (years)	760/-	51.4±11.8	-	
Self-reported family history of diabetes (%)	756/-	56.9	-	
BMI at age 20 (kg/m^2^)	577/162	22.9±4.2	22.6±2.8	0.706
Current BMI (kg/m^2^) at the time of examination	760/693	24.6±4.4	22.8±3.2	<0.0001
Maximum lifetime BMI (kg/m^2^)	760/693	27.7±4.8	24.8±3.2	<0.0001
Age at maximum life time BMI (years)	760/693	42.6±13.7	47.7±16.5	<0.0001
Waist circumference (cm) (male)	334/292	87.6±10.7	85.3±7.9	<0.05
Waist circumference (cm) (female)	219/260	87.9±12.3	81.4±9.3	<0.0001
FPG (mmol/l)	700/666	7.57±2.02	5.33±0.59	<0.0001
HbA1c (NGSP value) (%)	760/681	7.56±1.33	5.54±0.24	<0.0001
HOMA-β (%)^a^	460/659	38.9±34.2	61.4±42.4	<0.0001
HOMA-IR (mol・μU/l^2^) ^a^	460/659	2.25±1.79	1.25±0.82	<0.0001

Data are means ± SD.

* Pearson’s chi-square test.

^a^ HOMA-β and -IR were calculated in all participants except for those treated with insulin therapy.

### Anthropometric measurements and assessment of BMI history

The anthropometric measurements of individuals wearing light clothing and without shoes were conducted by well-trained examiners. Body height and weight were measured to the nearest 0.1 cm and 0.1 kg, respectively. The current BMI was calculated from these measurements. The waist circumference (WC) measurements were obtained at the end of normal expiration and were measured to the nearest 0.1 cm at the umbilical level using a flexible anthropometric tape. Each participant was questioned by a physician about their body weight (BW) history including their BW at the age of 20 years, their lifetime maximum BW, and the age at the time of their lifetime maximum BW. When ages and weights for the maximum BW and the BW at the age of 20 years were reported as a range, and not a single figure, the means of the minimum and maximum values were used for the analyses. If the participant’s BW at the time of the examination (Current BW) was greater than the self-reported maximum BW, the current BW was considered to be the lifetime maximum BW. To calculate the BMI_20y_ and the BMI_max_, we used the height measured at the time of the examination.

### Collection of clinical measurements and information except for anthropometric measurements

We obtained clinical information including the family history of diabetes and the age at diagnosis from self-reported data and medical records. We also examined the blood chemistry (including plasma glucose and HbA1c) during a fasting state. The HbAlc level was measured using high-performance liquid chromatography and was expressed as the international standard value, i.e., HbA1c (1.02 × Japan Diabetes Society [JDS (%)] + 0.25%), as defined by the JDS [27]. The homeostasis model assessment of insulin resistance (HOMA-IR) was calculated as previously reported [28].

All the study procedures conformed to the declaration of Helsinki and were approved by the Ethics Committee of the University of Toyama. Written informed consent was obtained from all the participants.

### Genotyping assay

Genomic DNA was extracted from the peripheral blood (QIAamp DNA blood kit; QIAGEN, Hilden, Germany). Genotyping of the SNP was performed using TaqMan SNP Genotyping Assays (Applied Biosystems, Foster City, CA, USA) or a multiplex-polymerase chain reaction (PCR)-invader assay, as described previously [25,29]. In this study, we selected four SNPs (rs8050136, rs9939609, rs1121980, and rs1558902) in the *FTO* locus; these were the most frequently analyzed SNPs at this locus in the Japanese population [16–20,30–34]. Previous studies have demonstrated that rs8050136, rs9939609, and rs1558902 are in high linkage disequilibrium (LD) with each other and were repeatedly associated with obesity or type 2 diabetes in different ethnicities [31,33,34]. First, we examined the LD among these three SNPs in our Japanese population and confirmed that these three SNPs were in absolute LD (*r*^2^ = 0.99) in our population (S2 Table). Then, since rs1558902 has been shown to have the strongest effect on susceptibility to obesity among 15 SNPs, including the above three SNPs, within the *FTO* gene in the Japanese population [33], we selected rs1558902 from among the three SNPs and performed an association study using rs1558902 and rs1121980 in this study. The success rates of these assays for rs1121980 and rs1558902 were 99.1% and 99.0%, respectively. The concordance rates for rs1121980 and rs1558902, based on duplicate comparisons in 221 T2D patients and 16 control participants, were 99.6% and 100%, respectively. No apparent deviations in the genotype distributions from Hardy-Weinberg equilibrium (HWE) were observed for all the SNPs (*P* ≥ 0.05) (Table 2).

**Table 2:** Genotype, allele frequencies, and HWE of 2SNPs within *FTO* gene in the present population.

SNP ID	Risk allele/non-risk allele	Genotype			Genotype			Risk allele frequency		HWE		Reported OR	Power (%)	Number of samples for 80% power
		T2D			CONT			T2D	CONT	T2D	CONT			
rs1121980	A/G	AA	AG	GG	AA	AG	GG	0.233	0.214	0.985	0.486	1.27^a^	82	1350
	number of subjects	40	271	443	26	241	419							
rs1558902	A/T	AA	AT	TT	AA	AT	TT	0.203	0.180	0.884	0.717	1.27^a^	78	1500
	number of subjects	29	249	477	19	208	456							

T2D: Type 2 diabetes, CONT; control.

HWE: Hardy-Weinberg Equilibrium.

CaTS power calculator, CaTS: http://www.sph.umich.edu/csg/abecasis/CaTS/).

The prevalence of type 2 diabetes was assumed to be 10%, ∞ = 0.05.

^a^ OR for T2D of rs9939609 reported by Frayling TM et al. (Science 316: 889–894, 2007)

Since little is known regarding the OR of rs1121980 and rs1558902 for T2D, and both SNPs and rs9939609 were found to be strong LD (*r*^2^>0.8) (S2 Table), we used the reported OR of rs9939609^a)^ for T2D to calculate the estimated power of rs1121980 and rs1558902.

### Statistical analysis

The statistical analyses were performed using JMP for Windows, Version 10.0 (SAS Institute, Cary, NC, USA). The normality of the distributions was checked using the skewed score, and variables with skewed distributions were logarithmically (natural) transformed in subsequent analyses. Differences in the clinical features of the case and control groups were examined using the Student *t*-test or Pearson chi-square test. The effect of BMI history on the T2D risk and the association of the *FTO* variants with the current or maximum BMI were examined by calculating the β values using a multiple linear regression analysis with adjustments for related co-variables.

We performed HWE tests according to the method described by Nielsen et al [35]. The allele-specific odds ratios (ORs) for T2D were calculated using logistic regression with or without adjustments for age, sex, and current or maximum BMI. A stepwise logistic regression analysis was carried out to evaluate the interaction test for sex*genotype. Quantitative trait analyses for HOMA-IR were performed using a multiple linear regression analysis with adjustments for age, sex, and current BMI. Results with *P* values < 0.05 were considered statistically significant.

LD analyses were performed with SNPAlyze version 8.0.2 software (Dynacom, Chiba, Japan). The power estimation for the present study to identify the association of previously reported SNP loci with T2D was performed using “CaTS power calculator for genetic studies” software (http://www.sph.umich.edu/csg/abecasis/CaTS/).

## Results

The clinical characteristics of the T2D patients and the non-diabetic control individuals are summarized in Table 1. The current BMI and BMI_max_ in the T2D group were significantly higher than those in the non-diabetic controls (*P* < 0.05), whereas the BMI_20y_ was not different between the two groups (Table 1). The mean ages at the time of examination and of the BMI_max_ in the T2D group were significantly younger than those in the non-diabetic controls (mean age: 64.6 ± 11.5 years vs. 66.5 ± 10.8 years, *P* < 0.001, mean age at BMI_max_: 42.6 ± 13.7 years vs. 47.7 ± 16.5 years, *P* < 0.001) (Table 1).

The BMI_max_ is reportedly associated more strongly with the onset of T2D than the current BMI measured at the time of examination [36–38]. Then, we next evaluated the associations of the various aspects of BMI history, such as the current BMI, BMI_max_, and BMI_20y_, with T2D using a multiple linear regression analysis with adjustments for related co-variables (Table 3). The results indicated that the BMI_max_ showed the strongest association with T2D among the BMI variables evaluated in either the sex-separated or sex-unseparated analysis (Table 3). The associations of the 2 SNPs with the current BMI and BMI_max_ are shown in Table 4. We did not observe a significant association between the variants within the *FTO* locus and the BMI variables in the un-stratified analysis. To evaluate the interaction between sex and SNP genotype statistically, we performed a stepwise multiple linear regression analysis (S3 Table). The results revealed a significant effect of the sex*SNP (rs1121980 and rs1558902) genotype interaction on both the current BMI and the maximum BMI (*P* < 0.05) (S3 Table). Therefore, we performed a stratified analysis according to sex and the SNPs were significantly associated with BMI_max_ only in men (βln-BMI_max_ for rs1121980 = 0.022, SE = 0.009, *P* = 0.011; βln-BMI_max_ for rs1558902 = 0.024, SE = 0.009, *P* = 0.008), but not in women (Table 4).

**Table 3:** Association of the various aspects of BMI history with type 2 diabetes.

Sex	index	β	S.E.	*P*-value	co-variables
Men and Women					age and sex
	BMI at age 20	0.072	0.098	0.462	
	Current BMI	0.646	0.080	1.3×10^−15^	
	Lifetime maximum BMI	1.094	0.080	1.0×10^−25^	
Men					age
	BMI at age 20	0.188	0.124	0.129	
	Current BMI	0.381	0.121	0.002	
	Lifetime maximum BMI	1.058	0.116	6.4×10^−19^	
Women					age
	BMI at age 20	0.339	0.155	0.030	
	Current BMI	0.869	0.105	7.5×10^−16^	
	Lifetime maximum BMI	1.148	0.110	1.2×10^−23^	

The results of linear regression analysis with adjustment for co-valuable are presented.

Various aspects of BMI are log-transformed for the analyses.

**Table 4:** Association of 2 SNPs with current and lifetime maximum BMI in control and case subjects.

SNP ID	Risk allele^a^	Sex	Current BMI^b^		Maximum BMI^b^	
			β (SE)	*P* value	β (SE)	*P* value
rs1121980	A	All	-0.004(0.007)	0.505^c^	0.006(0.007)	0.368^e^
		Men	0.008(0.009)	0.388^d^	0.022(0.009)	0.011^f^
		Women	-0.017(0.011)	0.117^d^	-0.014(0.010)	0.166^f^
rs1558902	A	All	-0.003(0.008)	0.641^c^	0.006(0.007)	0.392^e^
		Men	0.011(0.009)	0.236^d^	0.024(0.009)	0.008^f^
		Women	-0.019(0.012)	0.114^d^	-0.017(0.011)	0.111^f^

^a^ Risk allele reported in the previous reports.

^b^ values are log-transformed for the analyses.

^c^ Results of logistic regression analysis with adjustment for age, sex, and disease status are shown.

^d^ Results of logistic regression analysis with adjustment for age and disease status are shown.

^e^ Results of logistic regression analysis with adjustment for sex, age at the time of lifetime maximum BMI, and disease status are shown.

^f^ Results of logistic regression analysis with adjustment for age at the time of lifetime maximum BMI, and disease status are shown.

All; n = 1453.

Men; n = 789.

Women; n = 664.

Next, we examined the associations of the *FTO* variants with T2D with adjustments for age and sex as model 1 (Table 5). In addition, we added the current BMI or the maximum BMI as a covariate and examined the associations, as shown in models 2 and 3, respectively (Table 5). We did not observe a significant association between the *FTO* variants and T2D in an un-stratified analysis, but in sex-stratified analyses, rs1558902 was associated with T2D only in men when examined using a logistic regression analysis with adjustments for age (OR = 1.32 [95% CI, 1.02–1.72], *P* = 0.037) (Table 5). The association of the SNP with T2D remained after adjustments for the current BMI and age (*P* = 0.049), whereas the T2D association of the SNP was no longer significant after adjustments for age and BMI_max_ (*P* = 0.155). In women, the polymorphisms were not associated with T2D with or without adjustments for related co-variables.

**Table 5:** Association results between 2 SNPs and type 2 diabetes.

SNP ID	Model 1		Model 2		Model 3		
	OR (95% CI)	*P* value	OR (95% CI)	*P* value	OR (95% CI)		*P* value
Men and Women							
rs1121980	1.11	0.258	1.12	0.213	1.11		0.282
	(0.93–1.33)		(0.94–1.35)		(0.92–1.35)		
rs1558902	1.15	0.154	1.16	0.134	1.15		0.170
	(0.95–1.39)		(0.96–1.41)		(0.94–1.42)		
Men							
rs1121980	1.21	0.127	1.19	0.154	1.12		0.382
	(0.95–1.55)		(0.94–1.53)		(0.87–1.45)		
rs1558902	1.32	0.037	1.30	0.049	1.22		0.155
	(1.02–1.72)		(1.00–1.69)		(0.93–1.61)		
Women							
rs1121980	1.00	0.991	1.07	0.643	1.10		0.513
	(0.76–1.31)		(0.81–1.42)		(0.82–1.47)		
rs1558902	0.98	0.874	1.05	0.735	1.09		0.577
	(0.73–1.30)		(0.78–1.42)		(0.80–1.49)		

Model 1: adjusted for age with or without sex in sex-combined analysis or sex-stratified analysis, respectively.

Model 2: adjusted for log-transformed current BMI and all the variables included in Model 1.

Model 3: adjusted for log-transformed maximum BMI and all the variables included in Model 1.

## Discussion

In this study, we examined the associations of SNPs within the *FTO* with susceptibility to T2D in a Japanese population and found that the SNP was associated with T2D through their effects on the BMI_max_ in men.

Whether the effects of *FTO* variants on the susceptibility to T2D are mediated by their effects on obesity/adiposity remains controversial [1,9–20,39,40]. In addition, we thought that analyses using the “current BMI”, as conducted in many previous studies [1,9–17,19,20], might underestimate the influence of obesity on the association of *FTO* variants with T2D, and that the BMI_max_ before or at the time of T2D onset, rather than the“current BMI”, might be a better variable for understanding the precise mechanisms of the effects of *FTO* variants on the susceptibility to T2D for the reason mentioned in the Introduction section. Indeed, the BMI_max_ showed a stronger association with T2D than the current BMI in this study as previously reported [36–38], and, only in men, the association of the *FTO* variants with T2D was nominally significant when we used 'current BMI' and age as co-variables, whereas the association was no longer significant when we used BMI_max_ and age as co-variables (Table 5). These results indicated that the effect of *FTO* variants on T2D susceptibility in Japanese men is mediated through an effect that increases the BMI_max_.

In this study, we did not observe any associations of *FTO* variants with BMI variables or with HOMA-IR (Table 4 and S4 Table). To calculate the estimated power in this study, since little is known regarding the OR of rs1121980 and rs1558902 for T2D, and both SNPs and rs9939609 were found to be strong LD (*r*^2^>0.8) (S2 Table), we used the OR of rs9939609 for T2D (OR = 1.27) reported by Frayling TM et al [1] (Table 2). When using the reported OR of rs9939609 [1] at the alpha level of significance of 0.05, the estimated powers in this study were 82% and 78% for rs1121980 and rs1558902, respectively, in the sex-combined analysis (Table 2). However, when stratified according to sex, these powers decreased to about 50% for both rs1121980 (men, 56%; women, 49%) and rs1558902 (men, 52%; women, 48%). Taken together, although it has been reported that the effects of *FTO* variants on obesity risk may be reduced in older individuals like our participants in this study [41,42], an insufficient study power because of the smaller sample size in this study may be a principal cause of the discrepancy between this study and several previously reported studies examining European populations [1,2,5,6,39,40,42–45] and the marginal association of *FTO* SNPs with T2D after adjusting for the current BMI (Table 5).

In this study, *FTO* variants were significantly associated with BMI_max_ only in men, but not in women. A few reports have shown a sex-specific effect of *FTO* variants on conferring susceptibility to obesity/adiposity [43,44,46–49], and some reports suggest that differences in the fat distribution and/or physical activity between men and women might affect the effects of *FTO* variants on obesity/adiposity [44–46,49]. In addition to the possible sex-specific effects, the larger sample size of men compared with women and the difference in the case/control ratio between men and women (Table 1) might lead to gender differences in the association between rs1558902 and T2D or BMI_max_.

In addition to the insufficient study power for this study, other limitations should also be taken into consideration. A potential collider bias might have influenced our findings for the following reasons. It has been well established that, for example, the waist-to-hip ratio (WHR) and the waist circumference (WC) are associated with the BMI. Aschard et al. showed that when examining genetic effects on the WHR and the WC with adjustments for the BMI, such adjustments for associated covariates, i.e., colliders, might lead to false-positive findings [50]. Similar to the findings of this previous report, since *FTO* SNPs have been reported to be associated with BMI, a marker of obesity, and obesity has been reported to be a strong risk factor for T2D, BMI could be considered as a collider covariate for *FTO* SNPs and T2D. Therefore, our positive findings that *FTO* SNPs were associated with T2D after adjustments for the current BMI (*P* = 0.049) might have been skewed by a potential collider bias. In addition, the information on BW at the age of 20 years and the lifetime maximum BW was obtained using a self-reported questionnaire based on their recall; therefore, these values might not be sufficiently accurate and might have skewed the present findings, although long-term memory regarding weight has been shown to be sufficiently accurate in 50-year-old individuals [51] [52]. Moreover, information on how long each participant maintained his or her BMI_max_ is lacking, which may affect the association of obesity with the risk of T2D in this study. In addition, both BMI_20yr_ and BMI_max_ were estimated using "height measured at the time of the examination (current height)". However, these BMI estimates might be skewed as a result of stature lost due to aging [53] because the average age of our study participants were relatively older (~65) as shown in Table 1. To exclude the possibility, we calculated the estimated maximum height from the current height and age using the equations reported by Cline MG et al [54]. And then, we calculated the BMI_20yr_ and BMI_max_ using the height and re-analyzed the association between *FTO* SNPs and the BMI_max,_ and the association between *FTO* SNPs and T2D after adjustment for the BMI_max_ (S5 and S6 Tables). As a result, when we re-performed our analyses using the estimated maximum heights, we also obtained consistent results with our original findings using current height (Table 4 and S5 Table, Table 5 and S6 Table). Further study, such as a prospective study involving a larger sample size, is required to elucidate the precise mechanisms responsible for the association of *FTO* variants with susceptibility to T2D.

In conclusion, our results suggest that the effect of *FTO* polymorphisms on T2D susceptibility is mediated through an increase in the BMI_max_ before or at the time of diagnosis in Japanese men.

## Supporting Information

S1 Table Clinical characteristics of all subjects including type 2 diabetic subjects who had reached their lifetime maximum BMI (BMI_max_) after the time of diagnosis and control individuals without information regarding their BMI_max_. Data are means ± SD.* Pearson’s chi-square test. ^a^ HOMA-β and -IR were calculated in all participants except for those treated with insulin therapy.
(XLSX)

S2 Table Pairwise linkage disequilibrium (LD) for four SNPs (rs8050136, rs9939609, rs1121980, and rs1558902) in the *FTO* gene. Values of *r*^2^ for pairwise LD analysis in 760 type 2 diabetic subjects and 693 control subjects.
(XLSX)

S3 Table Stepwise multiple linear regression analysis for the effect of the sex*SNP genotype interaction on current BMI and maximum BMI (ln-transfomed). Age, sex, genotype (rs1121980 or rs1558902) and sex*SNP genotype were allowed to enter into the stepwise model. ^a^ When examining the effect of sex*SNP genotype interaction on maximum BMI, we used the age at the time of lifetime maximum BMI.
(XLSX)

S4 Table Association of 2 SNPs (rs1121980 and rs1558902) with HOMA-IR in control subjects and diabetic subjects without medication. Results of linear regression analyses. The effect size corresponds to the β-coefficient (SE) per copy of the type 2 diabetes risk allele and was calculated using a linear regression analysis. ^a^ n = 659 (adjusted for sex, age, and BMI), ^b^ n = 746 (adjusted for age, sex, BMI and disease status).
(XLSX)

S5 Table Association of 2 SNPs with current and lifetime maximum BMI in control and case subjects when analyzed using estimated maximum height† instead of current height. ^a^ Risk allele reported in the previous reports. ^b^ values are log-transformed for the analyses. ^c^ Results of logistic regression analysis with adjustment for age, sex, and disease status are shown. ^d^ Results of logistic regression analysis with adjustment for age and disease status are shown. ^e^ Results of logistic regression analysis with adjustment for sex, age at the time of lifetime maximum BMI, and disease status are shown. ^f^ Results of logistic regression analysis with adjustment for age at the time of lifetime maximum BMI, and disease status are shown. All; n = 1453, Men; n = 789, Women; n = 664. †Estimated maximum height was calculated from the height measured at the time of the examination (current height) using the transformation reported by Cline MG et al (Hum Biol. 1989; 61:415–425) as follows: men, estimated maximum height(cm) = current height (cm)+3.27651–0.16541×(age)+0.00209×(age)^2^; women, estimated maximum height(cm) = current height(cm)+5.13708–0.23776×(age)+0.00276×(age)^2^. The estimated maximum height was only used to calculate the maximum BMI.
(XLSX)

S6 Table Association results between 2 SNPs and type 2 diabetes after adjustment for covariates calculated from estimated maximum height† instead of current height. Model 1: adjusted for age with or without sex in sex-combined analysis or sex-stratified analysis, respectively. Model 2: adjusted for log-transformed current BMI and all the variables included in Model 1. Model 3: adjusted for log-transformed maximum BMI and all the variables included in Model 1. †Estimated maximum height was calculated from the height measured at the time of the examination (current height) using the transformation reported by Cline MG et al (Hum Biol. 1989; 61:415–425) as follows: men, estimated maximum height(cm) = current height (cm)+3.27651–0.16541×(age)+0.00209×(age)^2^; women, estimated maximum height(cm) = current height(cm)+5.13708–0.23776×(age)+0.00276×(age)^2^. The estimated maximum height was only used to calculate the maximum BMI.
(XLSX)
